# Untargeted Metabolomic Analysis and Chemometrics to Identify Potential Marker Compounds for the Chemical Differentiation of *Panax ginseng*, *P. quinquefolius*, *P. notoginseng*, *P. japonicus*, and *P. japonicus* var. *major*

**DOI:** 10.3390/molecules28062745

**Published:** 2023-03-18

**Authors:** Ruifeng Ji, Thomas Avery Garran, Yilu Luo, Meng Cheng, Mengyue Ren, Xiuteng Zhou

**Affiliations:** 1 State Key Laboratory of Dao-di Herbs, National Resource Center for Chinese Materia Medica, China Academy of Chinese Medical Sciences, Beijing 100700, China; 2 School of Chinese Materia Medica, Guangdong Pharmaceutical University, Guangzhou 510006, China

**Keywords:** *Panax*, ginseng, UPLC-QTOF-MS, metabolomics, ginsenosides, chemometrics, marker

## Abstract

The *Panax* L. genus is well-known for many positive physiological effects on humans, with major species including *P. ginseng*, *P. quinquefolius*, *P. notoginseng*, *P. japonicus*, and *P. japonicus* var. *major*, the first three of which are globally popular. The combination of UPLC-QTOF-MS and chemometrics were developed to profile “identification markers” enabling their differentiation. The establishment of reliable biomarkers that embody the intrinsic metabolites differentiating species within the same genus is a key in the modernization of traditional Chinese medicine. In this work, the metabolomic differences among these five species were shown, which is critical to ensure their appropriate use. Consequently, 49 compounds were characterized, including 38 identified robust biomarkers, which were mainly composed of saponins and contained small amounts of amino acids and fatty acids. VIP (projection variable importance) was used to identify these five kinds of ginseng. In conclusion, by illustrating the similarities and differences between the five species of ginseng with the use of an integrated strategy of combining UPLC-QTOF-MS and multivariate analysis, we provided a more efficient and more intelligent manner for explaining how the species differ and how their secondary metabolites affect this difference. The most important biomarkers that distinguished the five species included Notoginsenoside-R_1_, Majonoside R_1_, Vinaginsenoside R_14_, Ginsenoside-Rf, and Ginsenoside-Rd.

## 1. Introduction

Many plants from the ginseng genus (*Panax*) are regarded as valuable traditional medicine resources, as well as valuable herbs in the herb and supplement industry globally. They are well known for many positive physiological effects on humans and widely used in traditional medicine systems, as well as in the health food and supplement industry [1]. In China, the genus consists of six native and one introduced species (*P. quinquefolius*). Of these seven species, five are recognized as medicinal and officially recorded in the *Pharmacopoeia of the People’s Republic of China* (2020 Edition): *P. ginseng* C.A. Meyer (ginseng, Chinese ginseng, renshen, RS), *P. quinquefolius* L. (American ginseng, xiyangshen, XYS), *P. notoginseng* F. H. Chen (notoginseng, sanqi, SQ), *P. japonicus* C.A. Meyer (Japanese ginseng, zhujieshen, ZJS), *P. japonicus* var. *major* (Burkill) C.Y. Wu and K.M. Feng (zhuzishen, ZZS). In the global ginseng market, RS has the largest market share, followed by XYS, SQ, ZJS, and ZZS in the descending order of market share [2].

**Figure 1:**
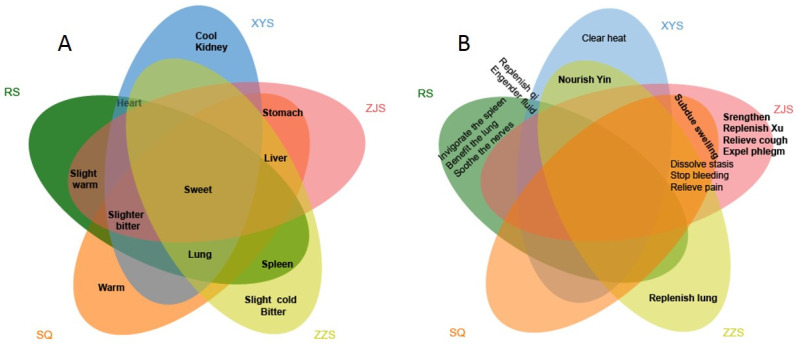
(**A**) Properties and (**B**) efficacy based on TCM theory for ginseng (RS), American ginseng (XYS), sanqi (SQ), zhujieshen (ZJS), and zhuzishen (ZZS).

As medicinal materials from the homologous species, they basically contain ginsenosides, polysaccharides, volatile oils, proteins, amino acids, organic acids, flavonoids, vitamins, and trace elements and other active ingredients. At the same time, their traditional efficacy and pharmacological activity are relatively similar. However, there are also some differences. For example, both RS and XYS have the effect of replenishing “*qi*” (a concept of traditional Chinese medicine, which is the energy of movement that reaches the skin externally and the organs internally, maintaining vitality) and producing fluid. SQ, ZJS, and ZZS all have the effect of removing blood stasis, stopping bleeding, and relieving pain. RS has antioxidant effects, can improve immunity, is anti-fatigue and anti-tumor, and can regulate the nervous system and immune system. XYS can improve blood circulation, improve the nervous system, and regulate immune function. SQ has the function of protecting the cardio-cerebrovascular and nervous systems and being anti-tumor, anti-bacterial, and anti-inflammatory. ZJS can protect the liver and heart, reduce blood fat, resist fatigue, enhance immunity, and is anti-tumor. ZZS can be anti-inflammatory, is an analgesic, is anti-tumor, and can regulate the immune function of the cardiovascular and cerebrovascular systems. According to traditional Chinese medicine (TCM) theory, RS and XYS are herbs for replenishing qi, and RS is considered slightly warm, while XYS is considered cool [3]. SQ is sweet and slightly bitter, warm in nature, and has a special tropism to the liver and stomach. ZJS and ZZS have a sweet and bitter taste similar to that of SQ, and they show tropism activity in the liver and stomach similar to SQ; however, they have additional tropism activity in the lung and thus can be used to treat phlegm and stop coughing. In addition, ZZS is slightly cold in nature and thus has additional efficacy in nourishing the lung-yin [4]. By integrating the information about the properties (nature, taste, and organ-specific channel tropism), as well as indications of RS, XYS, SQ, ZJS, and ZZS into a Venn diagram (Figure 1), we created a visual means of identifying the commonalities and differences in these herbs according to their traditional properties [5].

The phytochemistry and biological activities of *Panax* has been widely investigated. Although different species of *Panax* have diverse properties and indications in the TCM system, most literature presume that saponins (also known as ginsenosides) are the major active ingredients [6]. The current literature available regarding the metabolic difference studies of the same parts among different *Panax* species [7,8,9], the different parts [10] or ages [11,12] of the same species, and their combination demonstrates that ginsenosides have great potential when differentiating various species within the ginseng genus. The content and composition of ginsenosides that belong to the protopanaxadiol (PPD)-, protopanaxatriol (PPT)-, ocotillol (OCT)-, and oleanane (OA)-types; the C-17 side-chain-varied; the malonylated; and the others saponins vary widely in different ginseng species and in different parts of the plants [13]. Discrimination of the differences in the chemical composition among the five most commonly used species of *Panax* could inspire new research into potentially novel clinical applications of these species [14,15]. In contrast to the three most important *Panax* species (*P. ginseng*, *P. quinquefolius,* and *P. notoginseng*) covering very extensive studies, *P. japonicus* and *P. japonicus* var. *major* have far less literature support, particularly for the differences in their metabolome [16,17]. Characterizing the chemical components can ensure the correct use [18].

A key segment of the modernization research for TCM is the clarification of the chemical compositions for each species used [19]. The development of powerful and feasible analytical methods capable of the comprehensive deconvolution of plant metabolites that are from similar species is currently an important topic in the field of analytical chemistry [20,21]. Ultra-high performance liquid chromatography (UPLC) combined with quadrupole time-of-flight tandem mass spectrometry (QTOF-MS) is the most popular approach for the systematic multi-component characterization of an herb or a bio-sample, and UPLC-QTOF-MS profiling combined with chemometrics can render the untargeted metabolomics analysis suitable for discovering potential chemical markers for the authentication and quality evaluation of easily confused herb [11,22,23]. Yoon et al. (2022) reported the characterization of a total of 62 saponins from the geographical origin discrimination of *P. ginseng* by UPLC-QTOF-MS with multivariate analysis [24]. The establishment of a more powerful untargeted metabolomics platform can offer useful insights into the evaluation of the properties of medicinal plants, including easily confused species with similar pharmacological activity. 

Untargeted metabolomics can detect the dynamic changes of all small-molecule metabolites before and after stimulation or disturbance in cells, tissues, organs, or organisms without bias, screen differential metabolites through bioinformatics analysis, and conduct pathway analysis of differential metabolites to reveal the physiological mechanism of their changes. Targeted metabolomics is the study of a specific class of metabolites, used for the discovery and quantization of differential metabolites, and the in-depth research and analysis of subsequent metabolic molecular markers, which play an important role in disease research, animal model validation, biomarker discovery, disease diagnosis, drug development, plant metabolism, and other studies. Targeted metabolomics usually have several advantages over untargeted metabolomics, both in terms of sensitivity and specificity, as well as quantitative data processing.

In our case study, we presented an integral strategy of combining untargeted metabolomics with multivariate analysis and applied it to simultaneously differentiate five *Panax* species. PCA, PLS-DA, and OPLS-DA are all dimensionality reduction methods. They are used to look for a small number of principal components and to explain major changes in the data. We found that magnitudes were measured by the VIP of the chosen principal components. Our data showed that the newly established method was precise and rapid for distinguishing the five chosen species of ginseng.

## 2. Results and Discussion

### 2.1. Optimization of a UPLC-QTOF-MS Approach for the Enhanced Profiling and Characterization of Metabolites Simultaneously from RS, XYS, SQ, ZJS, and ZZS 

**Figure 2:**
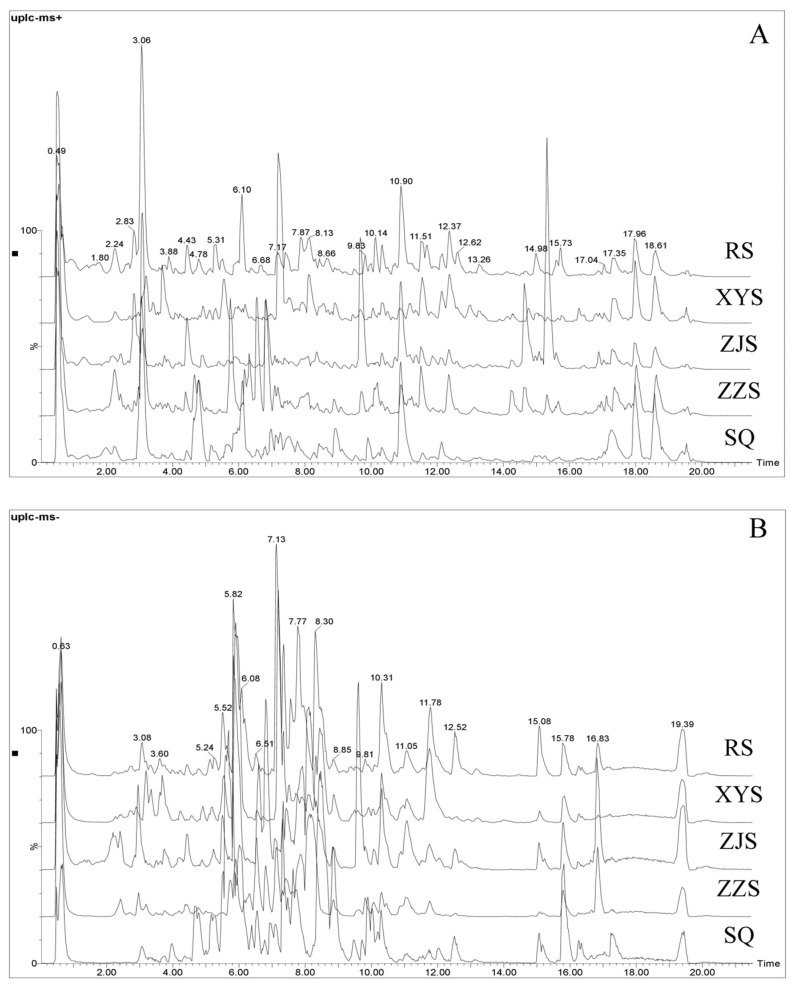
Base peak chromatograms for the representative samples of five Panax species. (**A**) In positive ion mode; (**B**) in negative ion mode.

The effects of the mobile phase system on the chromatographic peak and gradient elution on sample peak separation were investigated. The aqueous solution of formic acid containing 0 mol/L, 0.1 mol/L, 0.1 mol/L (containing 10 mmol/L ammonium formate), and 0.2 mol/L (containing 10 mmol/L ammonium formate) were investigated. The results showed that when gradient elution was applied with 0.2 mol/L formic acid solution (containing 10 mmol/L ammonium formate)–acetonitrile solution, both the peak shape and the separation effect were acceptable. Figure 2 shows the base peak chromatograms (BPC) at the optimized analysis conditions of the representative samples of five *Panax* species, which illustrate the metabolite differences among the five species. The similarity in the spectrograms of RS and XYS are obvious, while ZJS and ZZS have a similar composition. The essential characteristics are consistent with other literature [25,26]. The positive and negative ion data were interpreted so as to comprehensively and simultaneously characterize the five *Panax* species. After processing the chromatographic data, those listed as “Identified Compounds” were putatively identified using databases such as Pubchem and Massbank.

### 2.2. Multivariate Analysis for UPLC-QTOF-MS Results and Selection of Target Ion

**Figure 3:**
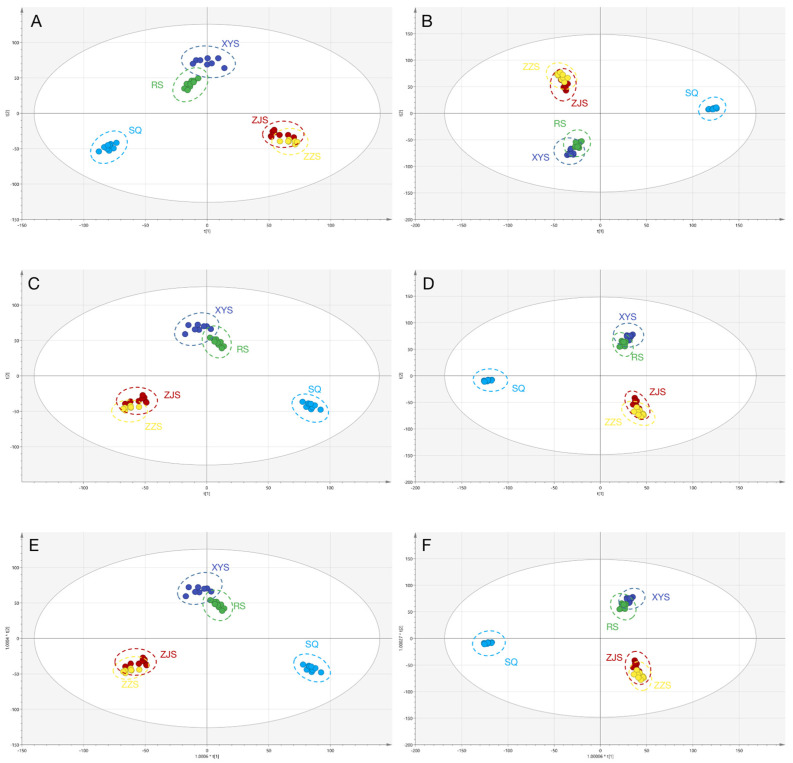
The PCA (**A**,**B**), PLS-DA(**C**,**D**), and OPLS-DA(**E**,**F**) score plots of five Panax species (RS, XYS, SQ, ZJS, and ZZS). (**A**,**C**,**E**) represents the score plots in positive ion mode; (**B**,**D**,**F**) represents the score plot in negative ion mode.

To obtain more information on the components of the five species, UPLC-QTOF-MS data was used for untargeted component analysis. Multivariate statistical analysis methods, such as unsupervised principal component analysis (PCA), supervised principal component analysis (PLS-DA), and orthogonal partial least squares discrimination analysis (OPLS-DA), were performed to identify the differences in metabolic profiles among the species. The quality of the model is dependent on the values of R^2^Y and Q^2^; when the values are higher (>0.9 and close to 1), the model is more reliable [27]. R^2^Y and Q^2^ represent the explanatory rate model and the forecast rate, respectively. The MS data of samples were statistically analyzed by PCA, PLS-DA, and OPLS-DA. The score plots of each revealed the factors that accounted for the largest variations and grouping tendencies. We found that the clustering degree of the samples in the PCA model indicated that the instrument was stable during this experiment in the positive and negative ion modes (Figure 3A,B). We also found signs of partial separation among the five groups. Using the PLS-DA method to analyze the metabolite profile of the *Panax* samples, the samples from RS, XYS, SQ, ZJS, and ZZS were clearly clustered and segregated into different groups scattering in different quadrants up to the 95% Hotelling T^2^ ellipse. There were significant differences among the five groups (ZZS is a variant of ZJS that considers partial overlap of results acceptable): [R^2^X (cum) =0.592, R^2^Y (cum) = 0.992, Q^2^ (cum) = 0.985] in positive ion mode, and [R^2^X (cum) =0.671, R^2^Y (cum) = 0.992, Q^2^ (cum) = 0.986] in negative ion mode (Figure 3C,D). It can be seen that R^2^Y and Q^2^ are both above 0.5 and close to 1, indicating the good reliability, good predictability, and no over-fitting for the PLS-DA model. These findings indicate that the PLS-DA model can be used to distinguish the five species. To obtain the greatest separation of metabolites, OPLS-DA was performed (Figure 3E,F). In the positive and negative ion modes, there was a clear separation among the groups. (ZZS is a variant of ZJS that considers partial overlap of results acceptable.) The samples from different species tended to cluster in a concentrated manner, with a high degree of aggregation and without any obvious intragroup differences. We found that the result of the OPLS-DA model was consistent with the PLS-DA model, and the Q^2^ of PLS-DA model was higher than the OPLS-DA model ([R^2^X (cum) = 0.592, R^2^Y (cum) = 0.992, Q^2^ (cum) = 0.977] in positive ion mode, and [R^2^X (cum) = 0.671, R^2^Y (cum) = 0.992, Q^2^ (cum) = 0.981] in negative ion mode). These findings indicated that the PLS-DA model had the highest discrimination and prediction rates (*p* < 0.05).

**Figure 4:**
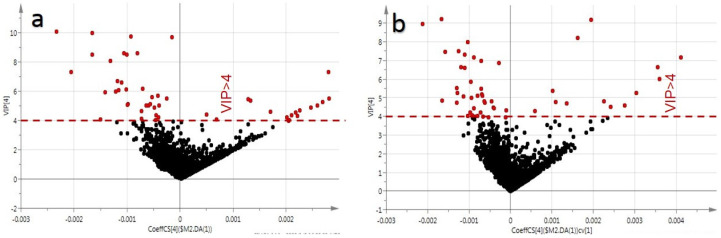
The VIP plots of five Panax species (RS, XYS, SQ, ZJS, and ZZS). (**a**) Represents the VIP plot in positive ion mode and (**b**) represents the VIP plot in negative ion mode.

**Figure 5:**
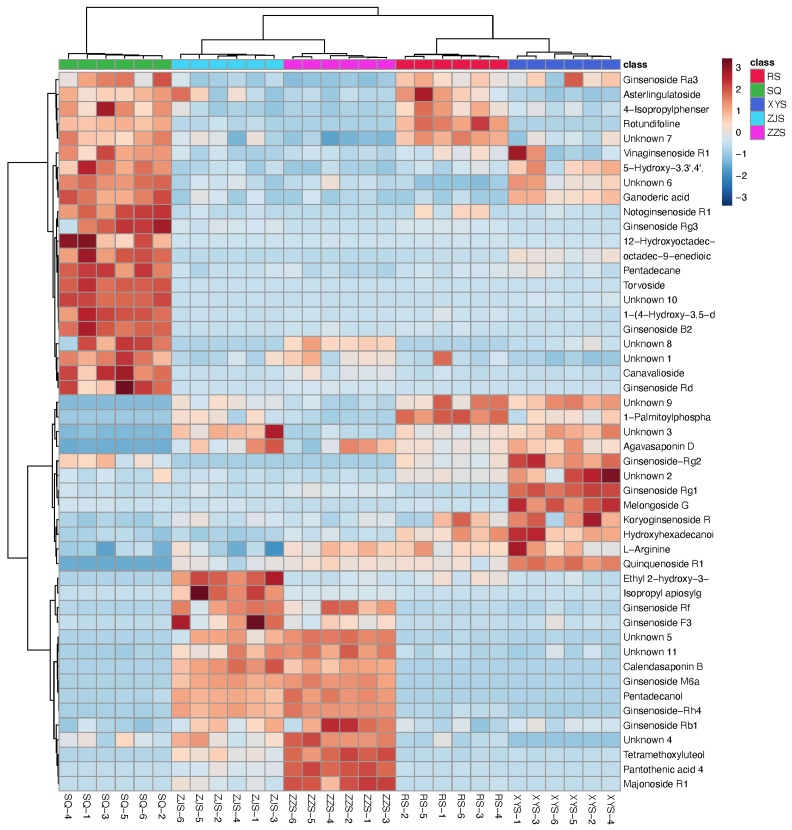
Heatmap visualization of metabolomics data with hierarchical clustering analysis (HCA). The red color represents the peak value that is relatively large; the blue color represents the peak value that is relatively small. The more similar the color, the more similar the peak value. The units in the abscissa axis represent the sample names and their groups; the panel on the right represents the different metabolites. The upper dendritic structure is clustered according to the degree of metabolite similarity across samples.

Variable importance in projection (VIP) is a weighted sum of the squares of the PLS loadings. To distinguish the most important metabolites among the species, *p*-values and VIP scores were used to screen for differential components (Figure 4). Given VIP > 4.0 and *p* < 0.05, a total of 49 potential biomarkers were preliminarily screened. The heatmaps of the component of significant difference among the species, as detected in the positive and negative modes, are illustrated in Figure 5. 

**Figure 6:**
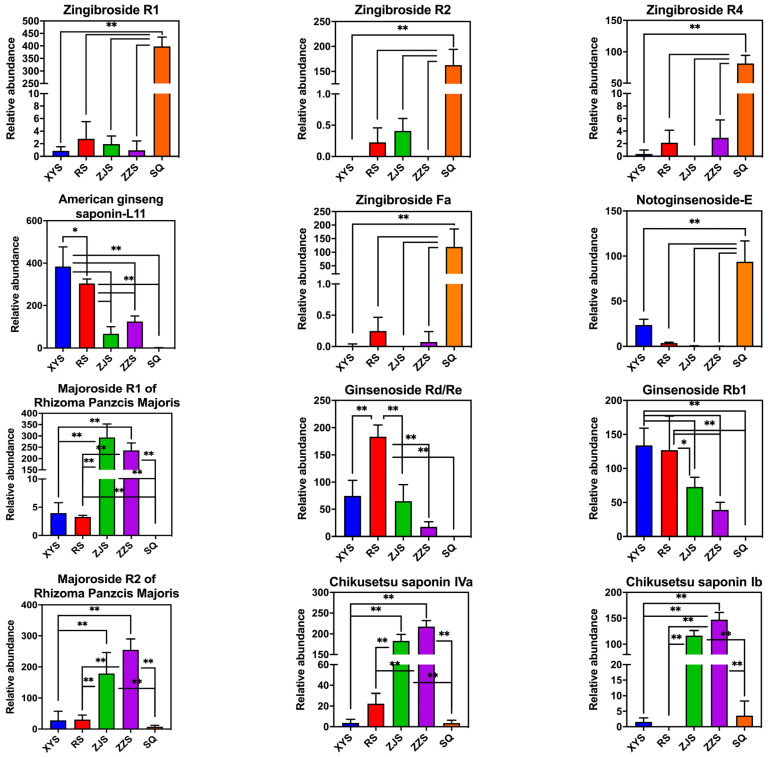
Bar charts illustrating the content differences in 12 important ginsenoside markers among RS, XYS, SQ, ZJS, and ZZS. (* *p* < 0.05, ** *p* < 0.01).

According to the screening results of potential markers, there were some significant differences in chemical composition between the five *Panax* species, including saponins, amino acids, and fatty acids. The primary differences were saponins (Table 1), such as t_R_ 6.80 and *m/z* 817.4781, identified as ginsenoside Re_5_ or ginsenjilinol, and t_R_ 8.45 and *m/z* 945.5336, identified as ginsenoside Rd or ginsenoside Re [28,29]. The levels of 12 ginsenosides showed significant differences in the five species. The spectral intensities of these 12 ginsenosides are presented as bar plots (Figure 6). These bar charts show the peak intensities for the target ion compounds, which vary significantly between species.

## 3. Materials and Methods

### 3.1. Reagents and Material

**Figure 7:**
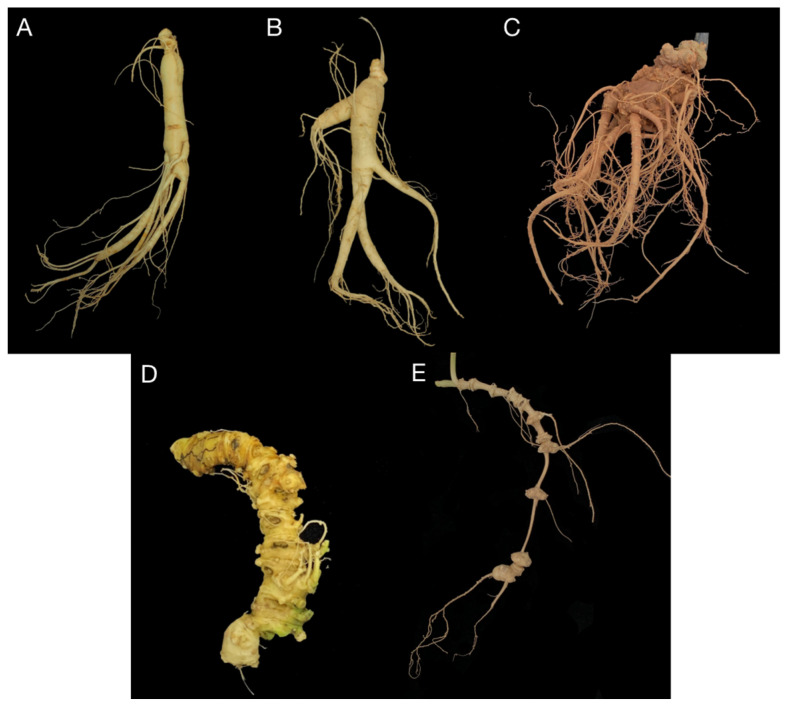
Appearance of *P. ginseng* (**A**), *P. quinquefolius* (**B**), *P. notoginseng* (**C**), *P. japonicus* (**D**), and *P. japonicus* var. *major* (**E**).

Acetonitrile, methanol (Merck, Darmstadt, Germany), leucine-enkephalin (LE, Sigma-Aldrich, St. Louis, MO, USA, No. L9133-50MG), formic acid (FA; CNW, Shanghai, China), and ammonium formate (AF; Sigma-Aldrich, MO, USA, No. 41470) were of HPLC grade. Deionized water was purchased from Guangzhou Watson’s Food & Beverage Co., Ltd. (Guangzhou, China). A total of five species of the officinal PSA herb genus *Panax,* including *Panax ginseng*, *P. quinquefolius*, *P. japonicus*, *P. japonicus* var. *major*, and *P. notoginseng,* were collected from wild or cultivated sources in China and the United States in 2018 (Figure 7).

### 3.2. Sample Preparation

The *Panax ginseng*, *P. quinquefolius*, *P. japonicus*, *P. japonicus* var. *major*, and *P. notoginseng* roots were powdered to a homogeneous size and sieved through a No. 60 mesh. The study involved 10 batches of RS, 8 XYS, 10 SQ, 8 ZJS, 10 ZZS. Ultrasonic extraction with 60% methanol at a solid-to-liquid ratio of 1:5 g·mL^−1^ for 1 h was used to extract the medicinal materials. Dried crude powder (0.3 g) was accurately weighed in a centrifuge tube, and then 1.5 mL of 70% methanol/water (*v/v*) solution was added. Then, the sample was processed using ultrasonic extraction for 45 min and cooled to room temperature. The sample solution was centrifuged at 12,000 rpm for 5 min at 20 °C, and the supernatant was stored at 4 °C and filtered through a 0.22 μm filter membrane before injection for UPLC analysis. 

### 3.3. UPLC/QTOF-MS Analysis

The ultrahigh-performance liquid chromatography by an Acquity UPLC system (Waters, Milford, MA, USA) was coupled with high-resolution MS analysis by a Micromass QTOF mass spectrometer (Waters, Manchester, U.K.). An Acquity UPLC BEH C_18_ column (2.1 mm × 50 mm, 1.7 μm) was used to perform the metabolite profiling, and 10 μL of each sample was injected into a gradient system at a flow rate of 0.3 mL/min. The mobile phase consisted of 0.2 mol/L formic acid and 10 mmol/L ammonium formate in water (A) and acetonitrile (B). The starting eluent was 2% B, and its proportion was held constant for 1 min, increased linearly to 10% from 1.0 to 2.0 min, to 25% from 2.0 to 5.1 min, to 32% from 5.1 to 6.1 min, to 38% from 6.1 to 8.1 min, held constant at 38% for 0.5 min, increased to 50% from 8.6 to 9.1 min, to 55% from 9.1 to 9.6 min, held constant at 55% until 12.5 min, increased to 70% from 12.5 to 14.0 min, to 100% from 14.0 to 16.1 min, held constant at 100% until 18.5 min, returned to 2% B at 18.5 min, and held constant until 20 min to equilibrate the column. This UPLC elution condition was optimized to detect the maximal number of metabolites in *P. ginseng*, especially to separate ginsenosides for identifying markers. The column was maintained at 40 °C. The mass spectrometer was equipped with an ESI source and operated in positive and negative ion modes. The MS conditions were as follows: capillary and cone voltages were adjusted to 2800 V and 20 V in positive ion mode but 3000 V and 20 V in negative ion mode, separately. The source and desolvation temperatures were maintained at 100 °C and 350 °C, respectively. The desolvation gas used was N_2_. The flow rate of desolvation gas and cone gas were at 500L/h and 50L/h, respectively. The scanning *m/z* range was 50 to 1500. Mass resolution > 10,000 FWHM (standard mode). To ensure that mass was measured accurately, leucine-enkephalin (10 μg/mL) was infused as the reference lockmass compound for real-time correction, and the [M+H]^+^ ion at 556.2771 Da and the [M-H]^−^ ion at 554.2615 Da were detected in the analysis. 

### 3.4. Multivariate Analysis

To evaluate the potential characteristic components of the five species of *Panax* in our study, the raw data of all samples were analyzed with the MassLynx application manager version 4.1 (Waters MS Technologies) [30]. The method parameters were as follows: retention time range, 0.3–17 min; mass range, 100–1500 Da; mass tolerance, 50 mDa; and noise elimination level, 6.00. For further analysis, a combination of retention time (t_R_) and mass data (*m/z*) from the detected peaks was assigned as temporary ID (t_R_-*m/z*), and the identifier of each peak for data adjustment was based on their chromatographic elution order of UPLC [31]. The identities of the variables chosen as biomarkers were based on *m/z*. The list was then applied for PCA, PLS-DA, and OPLS-DA using the Ezinfo software (Waters). Markers that differentiated the five groups were selected according to the variable importance in the projection values (VIP). The data matrix involving t_R_, *m/z*, and normalized peak area was exported into the SIMCA-P 14.0 software (Umetrics, Umea, Sweden) for chemometric analysis [32]. The compound with a VIP>4 was evaluated as the potential characteristic component from the positive and negative chromatogram [33]. The ANOVA value was lower than 0.05. Internal standard standardization was used.

## 4. Conclusions

Our study used a metabolomic fingerprinting approach based on discrimination by combining UPLC-QTOF-MS and multivariate analysis with the aim of discovering robust biomarkers for the authentication of the five most commonly used *Panax* species. The target compounds were selected from UPLC-MS screening results using multivariate analysis. Samples of the five *Panax* species were analyzed to create PCA, PLS-DA, and OPLS-DA models, which were compared with the UPLC-MS multivariate models. The resulting score plots showed good separation between the five species. In addition, the intensities of the target markers were compared with one another using bar plots to illustrate differences between the species. This is the first report to systematically compare the metabolome differences among these five important *Panax* species. The method elucidates highly efficient discrimination, especially considering the similarly of the samples, as required for authenticity testing. Moreover, with further optimization, this method could be used to analyze other plant materials to facilitate “chemical fingerprinting” and could be used as a new and important tool for the quality control of natural products derived from the *Panax* genus.

## Figures and Tables

**Table 1:** The information of potential chemical marker diagnostics for differentiating among RS, XYS, ZJS, ZZS, and SQ.

NO.	Ion Mode	Adducts	VIP	t_R_ (min)	Observed *m/z*	Error ppm	Formula	Identification	RS	XYS	ZJS	ZZS	SQ
1	+	M+H	5.43	0.49	175.1173	9	C_6_H_14_N_4_O_2_	L-Arginine	+	++	+	+	+
2	-	M-H	4.82	0.6	387.1074	3	C_20_H_20_O_8_	5-Hydroxy-3,3′,4′,7,8-pentamethoxyflavone	+	+	+	+	-
3	-	M-H	5.94	0.61	341.1003	8	C_19_H_18_O_6_	Tetramethoxyluteolin	+	+	++	+++	+
4	+	M+H-2H_2_O	5.95	0.64	360.1462	2	C_19_H_25_NO_8_	Ethyl 2-hydroxy-3-(3-indolyl)propanoate glucoside	+	++	+	+	++
5	+	M+NH_4_	5.02	2.41	372.1857	2	C_14_H_26_O_10_	Isopropyl apiosylglucoside	-	-	+	+	-
6	+	/	9.69	2.81	322.2084	/	/	Unknown 1	+	+	+++	+	-
7	+	/	8.56	2.94	386.1982	/	/	Unknown 2	-	-	++	-	-
8	+	/	9.72	3.07	363.169	/	/	Unknown 3	++	+	+	++	+++
9	+	/	4.35	3.68	400.2183	/	/	Unknown 4	-	+	-	-	-
10	+	M+CH_3_OH+H	5.67	3.82	579.2993	3	C_26_H_42_O_12_	Canavalioside	+	+	+	+	-
11	+	M+H	4.42	4.42	407.2007	14	C_22_H_30_O_7_	1-(4-Hydroxy-3,5-dimethoxyphenyl)-7-(4-hydroxy-3-methoxyphenyl)-3,5-heptanediol	+	-	+	++	+
12	+	M+NH_4_-H_2_O	6.08	4.65	381.1834	7	C_15_H_27_NO_10_	Pantothenic acid 4′-O-b-D-glucoside	-	-	-	-	+
13	+	M+H-2H_2_O	8.07	4.78	365.1871	0	C_22_H_28_N_2_O_5_	Rotundifoline	-	-	-	-	++
14	-	/	5.31	4.78	262.1171	/	/	Unknown 5	-	-	-	-	++
15	-	M-H	4.08	5.5	961.5382	0	C_48_H_82_O_19_	Ginsenoside M6a	-	-	-	-	+
16	-	M-H	7.73	5.63	931.5192	9	C_47_H_80_O_18_	Notoginsenoside R_1_	-	-	-	-	+++
17	+	M+H	8.57	5.73	817.4825	15	C_42_H_72_O_15_	Majonoside R_1_	-	-	+	++	-
18	-	M-H	4.91	5.83	799.4801	5	C_42_H_71_O_14_	Ginsenoside Rg_1_	-	-	-	-	++
19	-	/	7.13	5.88	991.5361	/	/	Unknown 6	++	+++	+	++	-
20	-	M+FA-H	4.91	6.05	845.4891	2	C_43_H_75_O_15_	Ginsenoside Rg_2_	-	-	-	+	+
21	+	M+NH_4_	7.29	6.06	904.5162	11	C_45_H_74_O_17_	Melongoside G	+	-	+	-	+
22	+	M+ACN+Na	6.57	6.31	580.3255	2	C_30_H_44_O_7_	Ganoderic acid	-	-	+	+	-
23	+	/	8.5	6.8	817.4781	/	/	Unknown 7	-	-	++	++	-
24	-	M+Hac-H	8.03	6.81	861.4695	18	C_41_H_70_O_15_	Vinaginsenoside R_14_	-	-	+++	++	-
25	+	M+2ACN+H	4.01	7	1259.6813	22	C_57_H_92_O_25_	Asterlingulatoside D	+	-	-	-	+
26	-	M-H	4.46	7.03	1239.6458	6	C_59_H_100_O_27_	Ginsenoside Ra_3_	-	-	-	-	+
27	+	M+2H	5.6	7.16	563.3038	4	C_54_H_92_O_24_	Koryoginsenoside Rg_2_	-	-	+	+	-
28	-	M+FA-H	8.19	7.16	845.4742	19	C_43_H_75_O_15_	Ginsenoside Rf	+++	+++	+	+	-
29	-	M-H	4.48	7.3	769.4709	5	C_41_H_70_O_13_	Ginsenoside F_3_	-	-	-	-	+
30	-	M+F	4.53	7.35	1193.5899	25	C_56_H_90_O_27_	Agavasaponin D	++	++	+	+	-
31	+	M+NH_4_	4.08	7.44	1126.6355	1	C_54_H_92_O_23_	Ginsenoside Rb_1_	-	+	-	-	+
32	-	M-H	4.06	7.65	783.4876	3	C_42_H_72_O_13_	Ginsenoside Rg_3_	-	-	-	-	+
33	-	M+Na-2H	5.31	7.82	925.4683	10	C_45_H_76_O_18_	Torvoside	-	-	+	+	-
34	+	M+NH_4_	5.11	8.13	1168.6486	1	C_56_H_94_O_24_	Quinquenoside R_1_	-	+	-	-	-
35	-	/	6.36	8.13	793.4239	/	/	Unknown 8	-	-	++	++	-
36	-	M+Hac-H	6.35	8.36	1031.5343	27	C_48_H_76_O_20_	Calendasaponin B	++	+	+	-	-
37	+	M+NH_4_	5.02	8.43	964.582	2	C_48_H_82_O_18_	Ginsenoside B2	-	+	-	-	+
38	-	M-H	7.22	8.45	945.5336	9	C_48_H_81_O_18_	Ginsenoside Rd	-	-	-	-	+++
39	+	/	4.69	8.66	1050.5853	/	/	Unknown 9	+	-	-	-	+
40	+	M+NH_4_	5.47	10.32	330.2622	5	C_18_H_32_O_4_	octadec-9-enedioic acid	+	+	-	-	+
41	+	M+NH_4_	5.35	10.88	316.2813	10	C_18_H_34_O_3_	12-Hydroxyoctadec-9-enoic acid	++	-	+	-	+
42	-	M-H+HCOONa	4.58	11.78	295.2235	7	C_15_H_32_O	Pentadecanol	+	+	-	-	-
43	+	/	5.51	12.1	622.3771	/	C_36_H_60_O_8_	Ginsenoside Rh_4_	+	+	-	-	+
44	+	M+H	4.07	12.37	496.3377	4	C_24_H_50_NO_7_P	1-Palmitoylphosphatidylcholine	+	+	+	+	-
45	+	/	6.69	14.64	378.2217	/	/	Unknown 10	-	-	++	++	-
46	-	M-H	4.33	15.07	271.2245	12	C_16_H_32_O_3_	Hydroxyhexadecanoic acid	+	-	-	-	+
47	+	M+H	9.95	15.32	380.236	7	C_23_H_29_N_3_O_2_	4-Isopropylphenserine	-	+	+++	++	-
48	-	M-H+HCOONa	5.25	15.79	279.2294	4	C_15_H_32_	Pentadecane	+	+	+	+	++
49	-	/	6.46	16.82	833.5103	/	/	Unknown 11	-	-	++	+++	-

+++: the content of the compound is very high, peak area ≥ 260; ++: the content of the compound is high, peak area ≥ 130 but <260; +: the content of the compound is low, peak area ≥ 10 but <130; - the content of the compound is very low or even none, peak area < 10.

## Data Availability

Data are contained within the article.

## Sample Availability

Samples of the research are available from the authors.

## Abbreviations List

VIP	variable importance in projection
RS	*P. ginseng*
XYS	*P. quinquefolius*
SQ	*P. notoginseng*
ZJS	*P. japonicus*
ZZS	*P. japonicus* var. *major*
TCM	traditional Chinese medicine
PPD	protopanaxadiol
PPT	protopanaxatriol
OCT	ocotillol
OA	oleanane
PCA	principal component analysis
PLS-DA	supervised principal component analysis
OPLS-DA	orthogonal partial least squares discrimination analysis
HCA	hierarchical clustering analysis
